# Exploring behavioral determinants of antimicrobial dispensing in drug retail outlets of Addis Ababa, Ethiopia: a mixed methods study

**DOI:** 10.1038/s41598-025-20558-w

**Published:** 2025-10-17

**Authors:** Oumer Sada Muhammed, Mirgissa Kaba Serbessa, Teferi Gedif Fenta

**Affiliations:** 1 College of Health Sciences, School of Pharmacy, Department of Social and administrative pharmacy, Addis Ababa University, Addis Ababa, Ethiopia; 2 College of Health Sciences, School of Public health, Department of Preventive medicine, Addis Ababa University, Addis Ababa, Ethiopia; 3 College of Health Sciences, School of Pharmacy, Department of Social and Administrative Pharmacy, Addis Ababa University, Addis Ababa, Ethiopia

**Keywords:** Antimicrobial resistance, Antimicrobial dispensing, Behavioral determinants, COM-B model, Behavior change wheel, Mixed-methods, Ethiopia, Health care, Medical research

## Abstract

Antimicrobial resistance (AMR) is a critical global health threat, exacerbated by inappropriate dispensing practices. Despite efforts to improve antimicrobial stewardship, behavioral determinants influencing antimicrobial dispensing remain understudied, particularly in low-resource settings like Ethiopia. This study was aimed to explore the behavioral drivers of antimicrobial dispensing among pharmacy professionals working at community drug retail outlets of Addis Ababa. This convergent, parallel mixed-methods study was conducted in three randomly selected sub-cities of Addis Ababa from March to May 2025. The quantitative component addressed the prevalence and predictors of dispensing behavior, surveying 240 pharmacy professionals selected via multi-stage sampling from 120 drug retail outlets. Data were collected using digital (Google Forms) and paper-based questionnaires, measuring TDF-based behavioral determinants and dispensing behavior. Participants were pharmacists and druggists with at least three months of dispensing experience, selected from private pharmacies and drug stores. Hierarchical multiple regression and confirmatory factor analysis were performed using SPSS and AMOS. The qualitative component explored contextual nuances through in-depth interviews with 16 professionals until thematic saturation, analyzed using Dedoose software with a thematic approach, without a predefined framework to allow free expression of feelings. Data quality was ensured through pre-testing, digital validation, and transcription cross-checking. Quantitative findings revealed inappropriate dispensing in 53.75% of cases (129/240). One-sample t-tests showed significant positive deviations for most TDF domains (p < .001), except environmental context (p = .642). Hierarchical regression indicated demographic and behavioral factors explained 62% of dispensing behavior variance, with age (β = 0.630, p < .001), female gender (β = 0.343, p < .001), and behavioral factors; (Professional Role and Self-Regulation: β = 0.456, p < .001; Emotional and Social Dynamics: (β = 0.359, p < .001)) as positive predictors, while Skills and Belief Systems (β=-0.431, p < .001) and experience (≥ 5 years) negatively predicted appropriate dispensing (β=-0.304, p < .001). Qualitative insights highlighted systemic barriers, including patient pressure, profit motives, and weak regulatory enforcement, which often overrode professional knowledge. Triangulation revealed that while pharmacists understood guidelines, workplace pressures and financial incentives drove non-compliance. Inappropriate antimicrobial dispensing was prevalent and significantly influenced by key determinants: longer experience negatively predicted appropriate dispensing, while female gender positively predicted it. Behavioral factors, particularly social and commercial pressures like patient demand and competition, were stronger drivers than knowledge alone.

**Supplementary Information:**

The online version contains supplementary material available at 10.1038/s41598-025-20558-w.

## Introduction

Antimicrobial resistance (AMR) is a top global public health threat that diminishes the efficacy of antibiotics, making infections harder to treat and increasing morbidity and mortality^1–4^. Addressing this challenge requires understanding its contributing factors, particularly human behavior in healthcare settings^2,5^. A key intervention is the appropriate use of antimicrobials, which depends heavily on responsible dispensing behavior^6–8^. Inappropriate practices like providing antibiotics without a prescription or incorrect dosing fuel AMR^9–11^. These are driven by factors like knowledge gaps, attitudes, and cultural context^12–14^, making an understanding of these behavioral determinants essential for effective stewardship. This research paper delves into the behavioral determinants of antimicrobial dispensing practices in Addis Ababa, Ethiopia, a setting where AMR rates are alarmingly high^15^ and antibiotic dispensing without a prescription remains a common occurrence^16^. The dispensing behavior of healthcare professionals, particularly pharmacists and pharmacy technicians, is a key area of concern. Understanding the factors that influence their decisions to dispense or withhold antibiotics is paramount to designing targeted interventions aimed at curbing inappropriate antimicrobial use. Central to this inquiry are questions surrounding the root causes of this behavior. Is it primarily driven by a lack of knowledge or competence regarding appropriate antimicrobial use guidelines? Are deeply ingrained attitudes, shaped by societal norms and previous training, playing a significant role? Are pharmacy professionals adequately equipped with the practical skills and procedural knowledge necessary to consistently practice responsible dispensing?

Existing literature on antimicrobial dispensing often focuses on epidemiological data, prevalence of antibiotic resistance^15,17,18^, and the impact of interventions aimed at improving prescribing practices. While these studies provide valuable insights into the overall problem of AMR, they often lack a comprehensive examination of the underlying behavioral factors driving dispensing practices. Previous research may highlight the importance of regulatory enforcement and address issues of access to and affordability of antibiotics^19–21^, but frequently neglects to delve into the nuanced behavioral and social factors that influence the decisions made by individuals on the frontlines of antimicrobial dispensing. This gap in understanding hinders the development of effective and sustainable interventions that address the root causes of inappropriate dispensing behavior.

This study aims to fill a critical knowledge gap by employing a theory-driven approach. By using established behavioral models, we can move beyond a simple description of the problem to a deeper, more structured analysis of the factors influencing antimicrobial dispensing. Specifically, this research will be guided by the Theoretical Domains Framework (TDF) and the COM-B model (Capability, Opportunity, Motivation, and Behavior). These frameworks will enable a systematic exploration of the determinants of antimicrobial practices at multiple levels, from the individual healthcare provider to the broader societal context^22^. This theoretical grounding will not only enhance the validity and generalizability of our findings but will also provide a robust foundation for developing targeted and effective interventions that address the root causes of inappropriate dispensing behavior.

## Methods

### Study setting and period

This study was conducted within the drug retail outlets of Addis Ababa, Ethiopia, a city with an estimated population of 3,854,863 across eleven sub-cities. There are 2,832 private health facilities, including 1,319 drug vendors and stores, and 42 Kenema pharmacies. To ensure an unbiased and representative sample of Addis Ababa’s diverse pharmacy landscape for exploring dispensers’ behavioral determinants, three sub-cities Nifas-Silk Lafto, Bole, and Akaki Kality were randomly selected using probability sampling. The study was conducted from March to May, 2025.

### Study design

This study employed a convergent, parallel mixed-methods design, an approach characterized by the simultaneous and independent collection of both quantitative and qualitative data. This design allows for the direct comparison and integration of findings from both data sets during the interpretation phase, aiming to provide a more comprehensive understanding of the research problem than either method could achieve alone.

### Study population

The study population encompassed all pharmacy professionals (including both pharmacists and druggists) actively engaged in antimicrobial dispensing roles within drug retail outlets across Addis Ababa.

### Sample size determination and sampling technique

The sample size for the quantitative component of this study, investigating antimicrobial dispensing behavior among pharmacy professionals, was determined using a single proportion formula. Based on a 95% confidence level, a margin of error of ± 5%, and an estimated proportion of 67.7% for non-prescription antimicrobial dispensing in Addis Ababa^10^, an initial sample size of approximately 153 participants was calculated. Considering that stratified sampling was employed, a design effect of 1.5 was applied, increasing the sample size to approximately 230 participants. Finally, accounting for a potential 10% non-response rate, the target sample size was adjusted to 253 pharmacy professionals.

A multi-stage sampling technique was utilized for participant recruitment. Initially, three sub-cities (Nifas-Silk Lafto, Bole, and Akaki Kality) were randomly selected among 11 sub-cities. From each of these sub-cities, an equal number of drug retail outlets (totaling 120 across the three) were chosen. From each selected outlet, at least two pharmacy professionals were recruited to participate. For the qualitative component of the study, 16 pharmacy professionals were included, and data collection continued until saturation of themes was achieved.

### Eligibility criteria

For this study, eligibility for participation was restricted to pharmacy professionals (either pharmacists or druggists) actively engaged in antimicrobial dispensing within private pharmacies and drug stores in Addis Ababa. A key inclusion criterion was a minimum of three months of experience in their current role, ensuring practical dispensing exposure. This study focuses exclusively on private drug retail outlets due to the distinct nature of their dispensing practices. We intentionally excluded public health facilities because over-the-counter (OTC) dispensing is uncommon in those settings. The determinants of dispensing behavior in private outlets where commercial incentives can directly conflict with regulatory guidelines are fundamentally different from those in public-sector facilities, which operate with stricter procurement and oversight mechanisms. Combining these two heterogeneous groups would confound our analysis and obscure the unique structural, economic, and motivational factors that influence dispensing behavior in each context.

Finally, only those professionals who were available during the data collection period and provided informed consent were included.

### Study variables

#### Dependent variable: dispensing behavior

Independent variables: demographic variables(age, gender, practice setting, experience and level of education); behavioral determinates measured using TDF domains (Knowledge, skill, social and professional role, emotion, environmental factors, intention, reinforcement, behavioral regulations, optimism, beliefs about consequences and beliefs about capabilities.

### Measurement of variables

The dependent variable, dispensing behavior, was initially measured using an 8-item tool, demonstrating good reliability with a Cronbach’s Alpha of 0.840. An Exploratory Factor Analysis (EFA) was conducted, revealing a Kaiser-Meyer-Olkin (KMO) value of 0.700, indicating good sampling adequacy. Bartlett’s Test of Sphericity was significant (χ2 = 1322.031, df = 28, *p* <.001), suggesting that the data was suitable for factor analysis. The EFA resulted in a two-factor structure comprising 6 items after the removal of poorly loading items. Subsequently, a Confirmatory Factor Analysis (CFA) was performed. The overall fit of the CFA model was acceptable with indices like RMSEA (0.07),CFI (0.913) and GFI (0.917) which were acceptable.

Behavioral determinants (independent variables) were measured using 35 items aligned with the 14 domains of the Theoretical Domains Framework (TDF), selected based on prior scoping review evidence. The measurement tool demonstrated strong reliability with a Cronbach’s Alpha of 0.896. Both face and content validity assessments were conducted. Construct validity was not further explored in this study, as the TDF has been extensively validated in previous research, confirming its underlying structure and theoretical coherence.

The qualitative data collection tool was initially developed through an extensive literature review and expert consultations. It was carefully crafted to investigate the complexities of antimicrobial prescribing practices and to enable meaningful integration with quantitative findings. The tool was prepared in Amharic, the local language, and all interviews were conducted in Amharic to ensure clarity and cultural nuance. The audio recordings were subsequently translated and transcribed verbatim into English for analysis. Unlike the theory-driven quantitative tool, the qualitative tool was intentionally designed without a fixed theoretical framework to allow participants to freely share their perspectives, capturing unexpected themes and contextual insights. Its semi-structured format used broad, non-leading questions to balance focus and flexibility, supporting an in-depth exploration of prescribing behaviors while aligning with study objectives. The tool prioritized neutral wording to reduce bias, comprehensive coverage of key practice dimensions, and the ability to probe for deeper insights. By avoiding theoretical constraints, it encouraged authentic, participant-driven responses that complemented the Theoretical Domains Framework (TDF)-based quantitative analysis, ensuring robust methodological rigor for effective mixed-methods integration.

### Data collection process

For the quantitative component, we employed a dual-mode survey distribution strategy, beginning with digital dissemination via Google Forms through email and Telegram to efficiently reach pharmacy staff with internet access. Recognizing potential digital barriers, we complemented this with on-site paper-based questionnaires administered by five trained data collectors, ensuring participation from those preferring traditional methods and enhancing sample representativeness. This combined approach successfully achieved our target sample size. For the qualitative investigation, three experienced researchers with backgrounds in pharmacy practice conducted in-depth interviews after receiving specialized training in qualitative techniques. Their expertise, coupled with structured debriefing sessions, ensured methodological consistency and generated rich, nuanced insights into dispensing behaviors.

### Data quality assurance

To ensure the reliability and validity of our findings on antimicrobial dispensing behaviors, we implemented rigorous quality control measures across both quantitative and qualitative data collection. For the survey component, we pre-tested the questionnaire with a sample of pharmacy staff to identify and resolve any ambiguities before full deployment. Digital validation rules were applied in Google Forms to minimize data entry errors and maintain accuracy. The paper-based surveys were similarly monitored, with trained data collectors verifying completeness at the point of collection.

For the qualitative interviews, we ensured methodological consistency through specialized training of researchers and periodic debriefing sessions. All audio recordings were meticulously transcribed and cross-checked against original recordings to guarantee textual fidelity, while researcher reflexivity was maintained throughout to account for potential biases.

### Data analysis

The data was analyzed using SPSS V. 27 and AMOS software. Descriptive statistics and hierarchical multiple linear regressions were performed in SPSS to identify behavioral determinants of dispensing, while AMOS was used for confirmatory factor analysis (CFA) to validate the measurement model.

Mean scores of each TDF domain (constructs) was calculated and a one sample t-test was employed against the mean score 3 to identify the deficient domains that has to be targeted for intervention.

A hierarchical multiple linear regression was conducted to identify the demographic and behavioral determinants of dispensing behavior (DBM). Preliminary assumption checks confirmed that multi-collinearity was not a concern after exploratory factor analysis (EFA) that reduced the TDF into three factor structure (Table 1), as evidenced by VIF values well below the common threshold of 5 (max VIF = 2.491).

**Table 1:** The reduced factor structure components after EFA.

Component	Items
Factor 1(Professional Role and Self-Regulation)	KMean, SPMean, INMean, GoMean, BRMean, ENMean
Factor 2(Emotional and Social Dynamics)	SIMean, EMMean
Factor 3(Skills and Belief Systems)	SKMean, BCMean, BCNMean, REMean

KMean: Knowledge, SPMean Social/professional role, INMean: Intention, GoMean: Goal, BRMean: Behavioral regulation, ENMean: Environmental context and resources, SIMean: Social influences, EMMean: Emotion, SKMean: Skill, BCMean: Belief about capabilities, BCNMean: Belief about consequences, REMean: Reinforcement,

Visual inspection of Q-Q plots and histograms suggested that the normality assumption for the residuals was largely met, with formal tests being sensitive and large sample size (240), justifying proceeding based on the Central Limit Theorem. The scatterplot of residuals also confirmed reasonable adherence to the linearity and homoscedasticity assumptions. The Durbin-Watson statistic of 1.983 indicated no violation of the assumption of independent errors.

We conducted a rigorous qualitative analysis using Dedoose software, employing a structured thematic approach. The analytical process began with verbatim transcription and translation of audio-recorded in-depth interviews (IDIs) with drug retail outlet personnel.

**Fig. 1:**
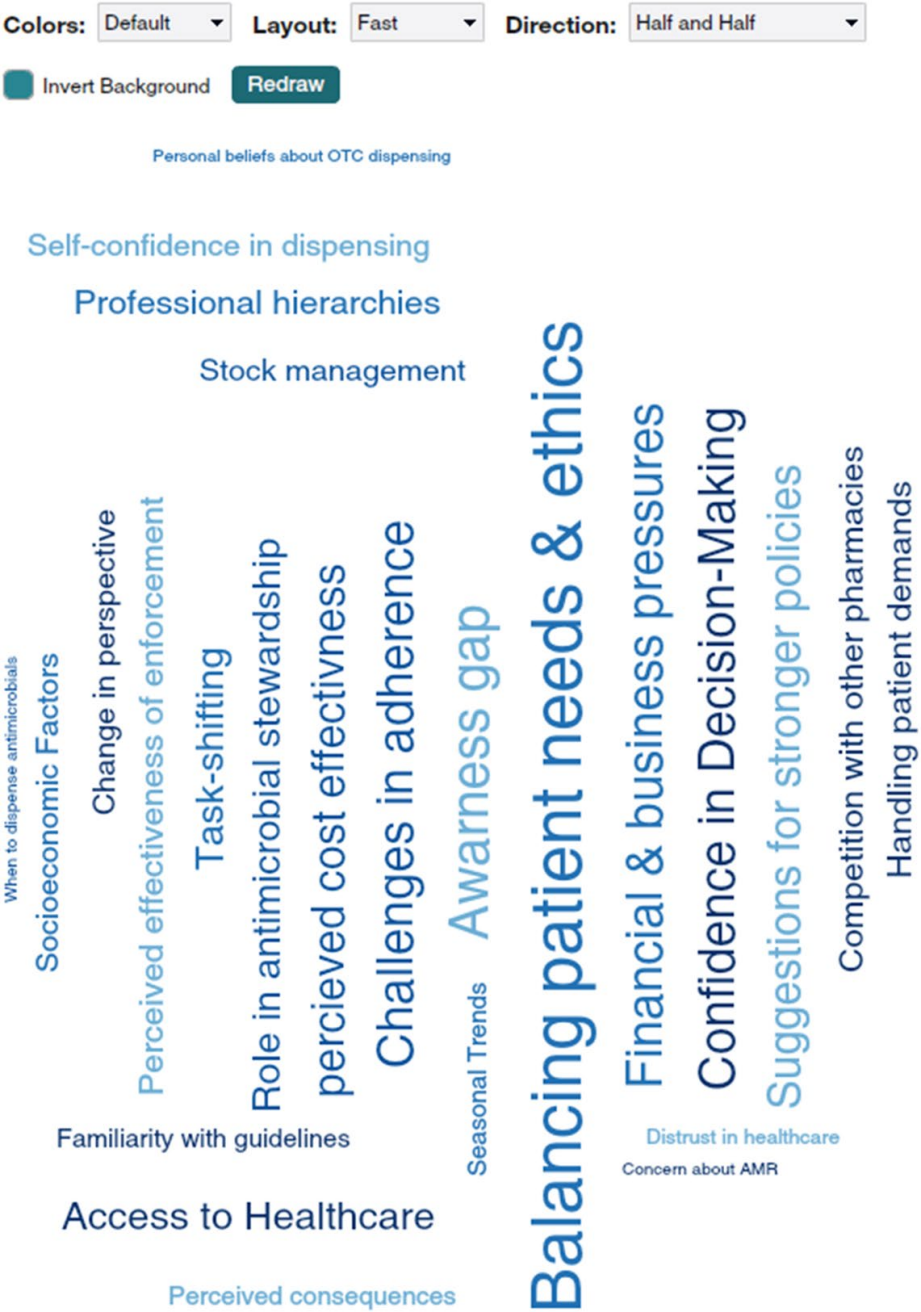
Dedoose’s packed code cloud visualizing code frequency.

After data cleaning and organization, we developed a preliminary codebook through an iterative process of independent coding by two researchers, followed by consensus-building discussions to refine code definitions and application rules (Fig. 1). Through constant comparative analysis, we synthesized the textual data into five key thematic categories that collectively explained antimicrobial dispensing behavior. These emergent themes were subsequently triangulated with quantitative survey findings to validate patterns and disparities. Finally, we mapped the behavioral determinants to the Behavior Change Wheel (BCW) framework to identify actionable intervention points specifically targeting capability, opportunity, and motivation barriers that could inform future antimicrobial stewardship initiatives in retail pharmacy settings.

### Operational definitions

Appropriate dispensing behavior: dispensing behavior score above the mean value (DB_ mean ≥ 3) was considered as appropriate dispensing behavior.

Deficient domain: a domain with near mean score, 3 or lower scores which indicates poor compliance with appropriate dispensing behavior.

### Ethical consideration

The study was approved by the Institutional Review Board (IRB) of Addis Ababa University, College of Health Sciences, and the research Ethics Review Committee of School of pharmacy. It was conducted in accordance with the Helsinki Declaration. Informed consent was obtained from participants. Confidentiality, neutrality, anonymity, accountability, and academic honesty were maintained throughout the study.

## Result

### Socio-demographic and professional characteristics

The survey yielded 240 completed responses from 253 distributed, achieving a 94.9% response rate. The study population had a mean age of 31 ± 7.0 years, with a gender distribution of 58.3% female (*n* = 140). Educational attainment showed the majority 67.9% had degrees or higher qualifications (*n* = 163). Most of the participants worked primarily in pharmacies (65.8%, *n* = 158). Experience levels were evenly distributed, with 50.4% having < 5 years (*n* = 121) and 49.6% possessing ≥ 5 years (*n* = 119) of practice experience(Table 2).

**Table 2:** Socio-demographic and professional characteristics of study participants (*N* = 240).

Variable	Frequency (%)
Age	Mean ± SD = 31 ± 7.0
Gender	
Male	100 (41.7)
Female	140 (58.3)
Level of education	
Diploma	77 (32.1)
Degree and above	163 (67.9)
Practice setting	
Drug store	82 (34.2)
Pharmacy	158 (65.8)
Year of experience	
Less than 5 years	121 (50.4)
5 years and above	119 (49.6)

### Mean scores and variability of behavioral determinants and dispensing behavior

Inappropriate dispensing behavior was observed in 53.75% (129/240) of cases. The analysis of mean scores of determinants influencing antimicrobial dispensing revealed significant variations across different factors. The highest mean score was observed for reinforcement (REMean: 4.55 ± 0.64), indicating strong perceived professional duties among dispensers, while the lowest score was for Emotion (EMMean: 2.74 ± 0.39). Other notable determinants included Social/professional role (SPMean: 4.23 ± 0.70) and Intentions (INMean: 4.25 ± 0.67). (Table 3).

**Table 3:** Mean scores and variability of behavioral determinants associated with antimicrobial dispensing Behavior.

Behavioral determinant	Mean value (± SD)
DBM	2.8648 ± 0.27
KMean	3.7569 ± 0.91
SKMean	4.1292 ± 0.59
SPMean	4.2271 ± 0.70
BCMean	3.9750 ± 0.77
DPMean	4.0097 ± 0.57
BCNMean	4.5542 ± 0.64
REMean	4.2500 ± 0.67
INMean	3.7125 ± 0.87
GoMean	3.5677 ± 0.82
MEMean	3.8979 ± 0.64
SiMean	2.7431 ± 0.39
EMMean	3.3000 ± 0.39
BRMean	3.4097 ± 0.69
ENMean	3.0181 ± 0.60

### One sample t-test result

The one-sample t-test was conducted to compare the sample means of multiple variables against a test value of 3. The results indicate that most variables (KMean, SKMean, SPMean, BCMean, OPMean, BCNMean, REMean, INMean, GoMean, MEMean, EMMean, BRMean) showed statistically significant differences from the test value (*p* <.001), with positive mean differences suggesting that their means were higher than 3. For example, BCNMean had the largest difference (Mean Difference = 1.554, 95% CI [1.473, 1.635]), indicating a strong deviation from the test value. In contrast, SIMean was significantly lower than 3 (Mean Difference = −0.257, 95% CI [−0.307, −0.207]). However, ENMean did not show a significant difference (*p* =.642, Mean Difference = 0.018, 95% CI [−0.058, 0.094]), suggesting that its mean is not statistically different from three (Table 4).

**Table 4:** One sample t-test result of behavioral determinates (*N* = 240).

One-sample test				
	Test value = 3			
	t	df	Sig. (2-tailed)	Mean difference
KMean	12.908	239	0.000	0.75694
SKMean	29.596	239	0.000	1.12917
SPMean	27.126	239	0.000	1.22708
BCMean	19.676	239	0.000	0.97500
OPMean	27.603	239	0.000	1.00972
BCNMean	37.833	239	0.000	1.55417
REMean	28.874	239	0.000	1.25000
INMean	12.696	239	0.000	0.71250
GoMean	10.692	239	0.000	0.56771
MEMean	21.586	239	0.000	0.89792
SIMean	−10.157	239	0.000	− 0.25694
EMMean	11.828	239	0.000	0.30000
BRMean	9.261	239	0.000	0.40972
ENMean	0.466	239	0.642	0.01806

### Behavioral determinates of antimicrobial dispensing

The hierarchical multiple regression analysis was performed in two blocks. **Model 1**, which included demographic variables (Age, Gend_Dum, Level of education, Practice setting, and Year of experience), significantly predicted DBM, accounting for 30.4% of the variance (R^2^ = 0.304, Adjusted R^2^ = 0.289, F (5, 234) = 20.429, *p* <.001). In this initial model, Age emerged as a significant positive predictor of DBM (B = 0.021,β = 0.554,t(234) = 6.820, *p* <.001), indicating that increasing age was associated with higher DBM (appropriate dispensing behavior). Being female (Gend_Dum) also significantly predicted higher DBM (B = 0.326,β = 0.591,t(234) = 9.015, *p* <.001). Conversely, Year of experience was a significant negative predictor (B = − 0.167,β=−0.307,t(234) = −4.415, *p* <.001), suggesting that more years of experience (i.e., 5 years and above) were associated with lower DBM. Level of education and Practice setting were not significant predictors in Model 1.

**Model** 2 incorporated the three behavioral determinant factor scores (Professional Role and Self-Regulation, Emotional and Social Dynamics, and Skills and Belief Systems), significantly enhancing the model’s explanatory power. This full model explained 62.0% of the variance in DBM (R2 = 0.620, Adjusted R2 = 0.607, F(8, 231) = 47.119, *p* <.001). The inclusion of the behavioral factors resulted in a substantial and statistically significant increase in the explained variance (ΔR2 = 0.316, F change​(3, 231) = 64.070, *p* <.001), indicating that these behavioral determinants uniquely accounted for an additional 31.6% of the variance in DBM beyond what was explained by demographics. In this final model, Age (B = 0.024,β = 0.630,t(231) = 9.848, *p* <.001) and Gender (female) (B = 0.189,β = 0.343,t(231) = 6.270, *p* <.001) remained significant positive predictors. Level of education became a significant positive predictor (B = 0.064,β = 0.110,t(231) = 2.294, *p* =.023), while Year of experience remained a significant negative predictor (B = − 0.165,β=−0.304,t(231) = −5.873, *p* <.001). Practice setting remained non-significant. Crucially, all three behavioral factors were highly significant predictors of DBM: Professional Role and Self-Regulation: (B = 0.124,β = 0.456,t(231) = 9.869, *p* <.001), Emotional and Social Dynamics: (B = 0.098,β = 0.359,t(231) = 7.164, *p* <.001), and Skills and Belief Systems : (B = − 0.117,β=−0.431,t(231) = −8.178, *p* <.001). These findings underscore the robust influence of both demographic characteristics and, more notably, the specific behavioral determinants on dispensing behavior (Table 5).

**Table 5:** Hierarchical multiple regression analysis of behavioral determinants influencing antimicrobial Dispensing, (*N* = 240).

Variables						
Model		Unstandardized coefficients		Standardized coefficients	t	P-value
		B	Std. Error	Beta		
1	(Constant)	1.889	0.129		14.694	0.000
	Age	0.021	0.003	0.554	6.820	0.000
	Gender	0.326	0.036	0.591	9.015	0.000
	Level of education	0.049	0.037	0.084	1.313	0.190
	Practice setting	− 0.026	0.037	− 0.045	− 0.706	0.481
	Year of experience	− 0.167	0.038	− 0.307	−4.415	0.000
2	(Constant)	1.952	0.103		19.006	0.000
	Age	0.024	0.002	0.630	9.848	0.000
	Gender	0.189	0.030	0.343	6.270	0.000
	Level of education	0.064	0.028	0.110	2.294	0.023
	Practice setting	− 0.006	0.030	− 0.010	− 0.187	0.851
	Year of experience	− 0.165	0.028	− 0.304	−5.873	0.000
	Emotional and social dynamics	0.124	0.013	0.456	9.869	0.000
	Professional role and self-regulation	0.098	0.014	0.359	7.164	0.000
	Skills and belief systems	− 0.117	0.014	− 0.431	−8.178	0.000

### Mapping of prioritized determinates with proposed interventions

Table 6 below systematically maps key dispensing challenges to theoretical domains and evidence-based behavior change techniques. It highlights critical gaps between knowledge and implementation, particularly the “experience paradox” where veteran pharmacists showed worse compliance despite training. Proposed interventions balance individual-level (e.g., peer mentoring) and system-level (e.g., digital diagnostics) solutions tailored to Ethiopia’s context. This structured approach moves beyond awareness-raising to address the complex motivational, social and environmental barriers underlying antimicrobial misuse.

**Table 6:** Behavioral determinates of antimicrobial dispensing prioritized for intervention development, mapped to the theoretical domains framework (TDF) and with the selected behavior change techniques (BCTs).

Priority factor	Key findings	TDF domain	Behavior Change Technique (BCT)	Proposed intervention
Knowledge-Practice Gap	Adequate knowledge but poor implementation	Knowledge	Instruction on how to perform behavior	Interactive workshops on interpreting partial prescriptions
Patient Pressure	“If I don’t dispense, another pharmacy will” mentality	Social Influences	Information about health consequences	Public awareness campaigns on antibiotic resistance
Financial Pressures	Revenue prioritized over AMR concerns	Reinforcement	Incentives	Performance-based rewards for appropriate dispensing
Regulatory Weaknesses	Punitive rather than supportive oversight	Environmental Context	Regulation	Collaborative inspection approach with education components
Experience Paradox	More experience linked to worse compliance	Skills/Beliefs	Social support	Peer mentoring programs pairing senior and junior pharmacists
Gender Differences	Female pharmacists showed better compliance	Social Identity	Credible source	Gender-sensitive training modules
Systemic Barriers	Incomplete prescriptions and lack of diagnostic support	Environmental Context	Restructuring environment	Digital prescription verification systems with diagnostic support

### Opportunities and challenges

Improving antimicrobial dispensing practices is crucial in combating antimicrobial resistance (AMR) and ensuring effective patient care. Key challenges were identified from the quantitative survey; widespread lack of awareness among patients and healthcare providers, frequent over-the-counter requests for antibiotics without prescriptions, overprescribing especially of higher-class antibiotics in private clinics, duplicate prescription of antimicrobials and luck of awareness of the resistance patterns of antimicrobials. However, significant opportunities were identified presence of CPD courses related to AMR, availability of online tools, and enhanced efforts to enforce prescription-only dispensing.

### Qualitative findings

This study employed thematic analysis to identify key behavioral determinates influencing antimicrobial dispensing decisions among pharmacy professionals working at community drug retail outlets. Through systematic examination of interview transcripts of pharmacy professionals, we identified recurring patterns in dispensing behaviors. Five major themes emerged from the analysis.

#### Theme I: knowledge and guideline adherence

Pharmacy professionals demonstrated a strong awareness of their role in antimicrobial stewardship, recognizing the importance of verifying prescriptions before dispensing. However, many reported challenges in assessing appropriateness when prescriptions lacked diagnoses, forcing them to rely on patient-provided information, which was often unreliable. One pharmacist noted, “We try to confirm if the medication is for the right person and disease, but when the diagnosis is missing, we rely on patient information which isn’t always accurate” (CPP005). While some participants referred to standard treatment guidelines (STGs) or online resources like Medscape for guidance, adherence was inconsistent due to time constraints and competing demands. Barriers to guideline use included prescriber non-compliance, particularly in private clinics, where higher-class antibiotics were often prescribed unnecessarily. As one pharmacist explained, “Private clinics prescribe high-class antibiotics for rapid relief, ignoring guidelines” (CPP003).

#### Theme II: OTC dispensing and patient pressure

Over-the-counter (OTC) dispensing of antimicrobials emerged as a significant concern, with pharmacists acknowledging its contribution to antimicrobial resistance (AMR). Many participants reported frequent patient demands for antibiotics without prescriptions, often based on past experiences. While some pharmacists resisted these requests through counseling and referrals, others admitted yielding to pressure, fearing loss of business. “Some say, ‘If I don’t dispense, another pharmacy will,’ so they give in” (CPP009).

#### Theme III: workplace and financial pressures

Workplace pressures, including high patient volume and profit-driven ownership structures, further complicated dispensing decisions. One pharmacist described the conflict between ethical practice and business demands: “Owners care about sales, not guidelines. We’re caught between ethics and keeping our jobs” (CPP003). The other pharmacist said, “When exhausted, you might skip verifying a prescription properly.” (CPP001).

#### Theme IV: prescriber influence and Task-Shifting

Interactions with healthcare providers, particularly prescribers in private clinics, also influenced dispensing behavior. Many pharmacists criticized private practitioners for unnecessarily prescribing higher-class and duplicate antimicrobials to attract patients, undermining stewardship efforts. “They start with high-class antibiotics to impress patients, leaving no options for severe cases” (CPP002). Opinions were divided on whether pharmacists should be authorized to manage minor infections like tonsillitis, with some advocating for task-shifting as a cost-effective solution and others fearing it would encourage self-medication.

#### Theme V: regulatory perceptions and recommendations

Regulatory approaches were widely viewed as ineffective, with participants criticizing punitive measures and calling for more educational interventions. “Inspectors just punish—they don’t teach. Behavior change is needed, not fines” (CPP0011). To address these challenges, participants recommended multifaceted interventions, including public awareness campaigns, prescriber accountability measures, and regulatory reforms that prioritize education over punishment. Strengthening guideline adherence through accessible resources and fostering collaboration between pharmacists and prescribers were also highlighted as key strategies. As one pharmacist emphasized, “We need systemic change better guidelines, prescriber accountability, and public awareness. Otherwise, resistance will keep rising” (CPP0014).

### Triangulation of the quantitative and qualitative findings

The study’s findings demonstrate strong convergence between quantitative and qualitative data, particularly in identifying the powerful social and financial determinants of dispensing behavior. Quantitatively, the factor “Emotional and Social Dynamics” was a highly significant positive predictor of appropriate behavior (β = 0.359, *p* <.001), which is vividly illustrated by the qualitative theme of patient pressure. The prevalent mentality that “If I don’t dispense, another pharmacy will” (CPP009) confirms that social influences are a critical driver, explaining why high perceived pressure correlates with inappropriate dispensing. This triangulation powerfully shows that the behavior is less a knowledge deficit and more a response to a competitive, commercial environment where ethical practice is economically penalized.

However, a critical area of divergence emerges around the role of knowledge. The quantitative survey found “Knowledge” scores were significantly above the test mean (Mean Diff. = 0.757, *p* <.001), suggesting adequate understanding. Conversely, the qualitative findings revealed a more nuanced “Knowledge-Practice Gap,” where participants reported significant challenges in assessing inappropriate prescriptions that lacked diagnoses, forcing them to rely on unreliable patient information. This divergence indicates that the quantitative measure may have captured theoretical knowledge, while the qualitative data uncovered the practical inadequacy of that knowledge in real-world, complex scenarios where prescriptions are incomplete or misleading.

## Discussion

The prevalence of inappropriate antimicrobial dispensing, as evidenced by the 53.75% rate in this study, reflects a critical impediment to achieving the strategic objectives of Ethiopia’s National Action Plan on Antimicrobial Resistance (NAP-AMR) 2021–2025^23^. This figure reveals both similarities and variations when compared to other research in Ethiopia and globally. While this study’s findings are lower than those of Belachew et al. (2022)^16^, who reported an 88% rate of non-prescription antibiotic dispensing in Ethiopian community drug retail outlets, they are higher than the 35.9% rate of improper antibiotic use identified in community surveys (Erku et al., 2017)^24^. Another Ethiopian study by Haile & Yabeyu (2022)^10^ found a 67.7% non-prescription dispensing rate, suggesting that regional or methodological differences may influence reported figures. Globally, the issue remains pervasive, with inappropriate antimicrobial prescribing ranging from 44% to 97% in low- and middle-income countries (Debela et al., 2022)^25^, indicating that structural and socioeconomic factors such as limited healthcare access and cultural self-medication practices play a significant role across contexts.

A seemingly contradictory demographic finding offers a crucial insight: while increasing age predicted better dispensing behavior, greater experience (≥ 5 years) predicted significantly worse behavior. This paradox suggests that tenure in a commercial environment may erode ethical commitment over time, an interpretation powerfully supported by our qualitative data. Veteran pharmacists described how financial pressures and profit-driven ownership structures gradually normalize non-compliance, with one stating, “Owners care about sales, not guidelines. We’re caught between ethics and keeping our jobs” (CPP003). This indicates that experience, without ongoing ethical reinforcement, may lead to cynicism and acquiescence to market demands.

Furthermore, the finding that female gender was a strong positive predictor of appropriate dispensing (β = 0.343, *p* <.001). This may be explained by culturally constructed professional identities; female pharmacists in Ethiopia may perceive their role more through a lens of caregiving and public health stewardship, potentially making them more resistant to commercial pressures that conflict with patient safety. Alternatively, it may reflect different practice styles or patient interactions. This finding, which contrasts with studies from Nigeria^16^, highlights the importance of context-specific social and cultural analyses in antimicrobial stewardship.

The most significant finding of our regression model is that behavioral factors accounted for over 31% of the variance in dispensing practices, far surpassing the contribution of demographics. This quantitative result is not a mere repetition of a statistic but a launch pad for interpretation, and it is vividly explained by the qualitative themes.

First, the strong positive association of Professional Role and Self-Regulation (β = 0.456, *p* <.001) confirms the value of training. However, pharmacists revealed that this capability is systematically undermined by Environmental Context and Resources. They described being unable to execute their knowledge due to incomplete prescriptions, lacking diagnostic support, and high patient volume, leading to situations where they “have to rely on patient-provided information which wasn’t always accurate” (CPP005). This triangulation clarifies that knowledge is a necessary but insufficient condition for appropriate dispensing.

Second, the potent role of Social Influences (quantified by the “Emotional and Social Dynamics” factor, β = 0.359, *p* <.001) was the most consistent theme across qualitative interviews. The pervasive fear of losing customers to competitors created a “spiral of compliance,” perfectly captured by the phrase “if I don’t dispense, another pharmacy will” (CPP009). This social pressure creates a powerful negative reinforcement loop that financially penalizes ethical behavior, making the economic incentive the dominant driver of practice. This makes it the most potent barrier to implementing the NAP-AMR^23^. The AMR’s regulatory approach must be complemented by strategies that alter the economic calculus of dispensing, such as public awareness campaigns to reduce patient demand (aligning with the NAP-AMR’s strategic objective of public awareness) and potential incentive models for compliant pharmacies.

Third, skills and belief systems presented a paradoxical picture. While stronger belief systems negatively correlated with appropriate dispensing (β=−0.431, *p* <.001), these professional ethics were frequently overridden by financial imperatives. This tension between ethical motivation and economic reality has been observed throughout Sub-Saharan Africa, with Tanzanian data showing 75% of pharmacies prioritizing revenue over AMR concerns^26^. Our finding that experience moderated this effect with veteran pharmacists demonstrating lower resistance to pressures suggests professional identity development as a potential intervention target. The gender difference we observed, with female pharmacists showing higher appropriate dispensing behavior scores (β = 0.343, *p* <.001), presents an important area for future research. While gender has not emerged as significant in Nigerian studies^27^, our finding may reflect unique workplace dynamics in Ethiopian pharmacies or culturally-specific patient-pharmacist interactions.

The study findings highlight significant gaps between policy and practice in antimicrobial stewardship. While regulations prohibit OTC antibiotic sales, weak enforcement and competing economic incentives create an environment where non-prescription dispensing remains common. Pharmacists criticized the current punitive regulatory approach, suggesting that inspectors focus too much on punishment rather than education and support. This aligns with national studies showing high rates of non-prescription dispensing despite pharmacists’ awareness of the regulations.

Our results both confirm and extend previous research in this area. While other African studies have emphasized knowledge gaps as a primary driver of inappropriate dispensing^28^, our mixed-methods approach reveals a more complex picture. Pharmacists in our study often possessed adequate knowledge but faced structural barriers to implementation, including pressure from patients and pharmacy owners. The debate around task-shifting whether to empower community pharmacists to manage minor infections also emerged as an important theme, with opinions divided on its potential benefits and risks.

### Mapping the findings with intervention strategies (BCW Framework)

Our comprehensive analysis using the COM-B model reveals specific behavioral diagnoses critical for designing targeted interventions to improve antimicrobial dispensing practices. Capability deficits are evident in the challenges pharmacists face in assessing prescription appropriateness without diagnoses and inconsistent adherence to guidelines despite awareness. This suggests a need for interventions enhancing psychological capability, such as providing readily accessible, real-time diagnostic support tools at the point of dispensing, and interactive training on interpreting partial prescription information^29^. To address opportunity barriers, specifically the lack of timely access to local resistance patterns and the overwhelming patient volume, interventions should focus on improving physical opportunity. This could involve developing user-friendly digital platforms that integrate local antibiogram data into dispensing workflows and optimizing pharmacy staffing models to reduce workload pressures^30,31^. The significant social opportunity challenges, including patient pressure for over-the-counter antibiotics and the influence of inappropriate prescribing by private clinics, necessitate multi-level interventions. These include public awareness campaigns (Education and Environmental Restructuring) to manage patient expectations and promote responsible antibiotic use, coupled with targeted communication strategies and feedback loops for prescribers (Persuasion and Modelling) to encourage guideline adherence and discourage the over-prescription of higher-class antimicrobials^32–34^. Finally, motivational conflicts, particularly the tension between ethical dispensing and financial pressures, highlight the need for interventions that reinforce reflective motivation. This could involve Incentivisation strategies that reward appropriate dispensing behaviors, Coercion through stricter enforcement of prescription-only regulations (coupled with Education on the rationale), and Enablement through professional development focused on ethical decision-making and strategies for navigating business pressures^35,36^. Furthermore, addressing the observed decline in appropriate dispensing with increased experience may require Modelling from senior, exemplary pharmacists and peer-to-peer Support interventions^37^. By systematically addressing these capability, opportunity, and motivation deficits through a combination of educational, environmental, and policy-level interventions, a more robust and sustainable change in antimicrobial dispensing behavior can be achieved.

### Limitations

The study has some limitations to consider. Our quantitative sample focused on urban pharmacists, which may limit generalizability to rural areas. The cross-sectional design prevents causal inferences, and self-report data may be subject to social desirability bias. Future research could benefit from longitudinal designs and inclusion of more rural participants.

## Conclusion

This study found that inappropriate antimicrobial dispensing is prevalent and is significantly predicted by specific demographic and behavioral determinants. Crucially, longer professional experience (≥ 5 years) was a significant negative predictor of appropriate dispensing, while female gender was a significant positive predictor. The quantitative finding that “Emotional and Social Dynamics” was a strong positive predictor (β = 0.359, *p* <.001), coupled with the qualitative theme of intense patient pressure and the fear of losing customers to competitors, confirms that the social and commercial environment is a primary driver of behavior, outweighing adequate theoretical knowledge.

### Recommendations

To effectively address the key determinants identified, we recommend a prioritized strategy targeting specific stakeholders. First, for the Ethiopian Food and Drug Authority (FDA): shift from a punitive to a supportive supervision model, where inspectors are trained to mentor rather than solely penalize, with a specific focus on guiding experienced dispensers to overcome entrenched non-compliant habits. Second, for professional associations and training institutions: mandate practical, continuous professional development that moves beyond theoretical knowledge to build essential skills in patient communication and managing pressure, thereby addressing the critical knowledge-practice gap. Finally, a multi-stakeholder effort led by the FDA and Ministry of Health is required: to launch public awareness campaigns reducing patient demand; these systemic interventions are essential to mitigate the overwhelming social and commercial pressures dispensers face, providing them with the legal and societal backing to adhere to guidelines.

## Supplementary Information

Below is the link to the electronic supplementary material.

Supplementary Material 1

Supplementary Material 2

## Data Availability

Data is provided within the supplementary information files.

## References

[1] Aslam, B. et al. AMR and Sustainable Development Goals: at a crossroads | Globalization and Health [Internet]. 2024 [cited 2025 Jul 2]. Available from: https://link.springer.com/article/10.1186/s12992-024-01046-8. 10.1186/s12992-024-01046-8 PMC11484313 39415207

[2] Alara, J. A. & Alara, O. R. An Overview of the Global Alarming Increase of Multiple Drug Resistant: A Major Challenge in Clinical Diagnosis | Bentham Science Publishers [Internet]. 2024 [cited 2025 Jul 2]. Available from: https://www.benthamdirect.com/content/journals/iddt/10.2174/1871526523666230725103902. 10.2174/1871526523666230725103902 37909431

[3] Ahmed, S. K. et al. Antimicrobial resistance: Impacts, challenges, and future prospects. *J. Med. Surg. Public. Health*. **2**, 100081 (2024).

[4] WHO. Antimicrobial resistance [Internet]. 2023 [cited 2025 Apr 24]. Available from: https://www.who.int/news-room/fact-sheets/detail/antimicrobial-resistance.

[5] Endale, H., Mathewos, M. & Abdeta, D. Full article: Potential Causes of Spread of Antimicrobial Resistance and Preventive Measures in One Health Perspective-A Review [Internet]. 2023 [cited 2025 Jul 2]. Available from: https://www.tandfonline.com/doi/full/10.2147/IDR.S428837. 10.2147/IDR.S428837 PMC10715026 38089962

[6] Abimbola, S. O., Otieno, M. A. & Cole, J. Reducing the use of antimicrobials as a solution to the challenge of antimicrobial resistance (AMR): approaching an ethical dilemma through the lens of planetary health. *Challenges* **12** (2), 23 (2021).

[7] Majumder, M. A. A. et al. Full article: Antimicrobial Stewardship: Fighting Antimicrobial Resistance and Protecting Global Public Health [Internet]. 2020 [cited 2025 Jul 2]. Available from: https://www.tandfonline.com/doi/full/10.2147/IDR.S290835. 10.2147/IDR.S290835 PMC7778387 33402841

[8] Batista, A. D. et al. Antibiotic Dispensation without a Prescription Worldwide: A Systematic Review [Internet]. 2020 [cited 2025 Jul 2]. (2079). Available from: https://www.mdpi.com/-6382/9/11/786. 10.3390/antibiotics9110786 PMC7694985 33171743

[9] Nahar, P. et al. What contributes to inappropriate antibiotic dispensing among qualified and unqualified healthcare providers in bangladesh? A qualitative study. *BMC Health Serv. Res.* **20**, 656 (2020). 32669092 10.1186/s12913-020-05512-y PMC7362537

[10] Haile, K. T. & Yabeyu, A. B. Dispensing Antibiotics without Prescription in Ethiopia | IPRP [Internet]. 2022 [cited 2025 Jul 1]. Available from: https://www.dovepress.com/knowledge-attitude-and-practice-of-pharmacy-professionals-against-disp-peer-reviewed-fulltext-article-IPRP. 10.2147/IPRP.S383709 PMC9717598 36465587

[11] Damisie, G., Hambisa, S. & Yimam, M. Over the counter sale of antibiotics at drug stores found in Mizan-Aman Town, Southwest ethiopia: A Cross-Sectional simulated client visit study. *J. Pharm.* **2019**, 3510659 (2019). 10.1155/2019/3510659 PMC6475547 31080686

[12] Al Masud, A. et al. Antibiotic dispensing practices in community pharmacies: implications for antimicrobial stewardship in resource-constrained settings. *Explor. Res. Clin. Soc. Pharm.* **19**, 100606 (2025). 40486980 10.1016/j.rcsop.2025.100606 PMC12142531

[13] Alhomoud, F. Antibiotics kill things very quickly - consumers’ perspectives on non-prescribed antibiotic use in Saudi Arabia | BMC Public Health | Full Text [Internet]. 2018 [cited 2025 May 20]. Available from: https://bmcpublichealth.biomedcentral.com/articles/10.1186/s12889-018-6088-z. 10.1186/s12889-018-6088-z PMC6192199 30326870

[14] Sun, R. et al. Non-biomedical factors affecting antibiotic use in the community: a mixed-methods systematic review and meta-analysis. *Clin. Microbiol. Infect.* **28** (3), 345–354 (2022). 34768017 10.1016/j.cmi.2021.10.017

[15] Woldu, M. A. Antimicrobial resistance in Ethiopia: current landscape, challenges, and strategic interventions | Discover Medicine [Internet]. 2024 [cited 2025 Jul 2]. Available from: https://link.springer.com/article/10.1007/s44337-024-00090-y.

[16] Belachew, S. A., Hall, L. & Selvey, L. A. Magnitude of non-prescribed antibiotic dispensing in ethiopia: a multicentre simulated client study with a focus on non-urban towns. *J. Antimicrob. Chemother.* **77** (12), 3462–3465 (2022). 36210768 10.1093/jac/dkac341 PMC9704429

[17] Berhe, D. F. et al. Prevalence of antimicrobial resistance and its clinical implications in ethiopia: a systematic review. *Antimicrob. Resist. Infect. Control*. **10**, 168 (2021). 34861894 10.1186/s13756-021-00965-0 PMC8642948

[18] Gemeda, B. A., Assefa, A. & Jaleta, M. B. Antimicrobial resistance in ethiopia: A systematic review and meta-analysis of prevalence in foods, food handlers, animals, and the environment. *One Health*. **13**, 100286 (2021). 34258373 10.1016/j.onehlt.2021.100286 PMC8260865

[19] Belachew, S. A., Hall, L. & Selvey, L. A. Handing out non-prescribed antibiotics is storing up trouble for the next generation! Unpacking multistakeholder views of drivers and potential solutions in Ethiopia. *BMC Health Serv. Res.* **23** (1), 830 (2023). 37550647 10.1186/s12913-023-09819-4 PMC10405379

[20] Edessa, D. et al. Drug providers’ perspectives on antibiotic misuse practices in Eastern ethiopia: a qualitative study. *BMJ Open.* **14** (8), e085352 (2024). 39209504 10.1136/bmjopen-2024-085352 PMC11404147

[21] Atnkut, B. et al. Assessment of inappropriate use of antibiotics and contributing factors in Awi Administrative Zone, Northwestern Amhara regional State, Ethiopia. *New Microbes New Infect.* **63**, 101557 (2025). 10.1016/j.nmni.2024.101557 PMC11728071 39807160

[22] Lou, A. et al. A guide to using the Theoretical Domains Framework of behaviour change to investigate implementation problems| Implementation Science [Internet]. 2017 [cited 2024 Dec 30]. Available from: https://link.springer.com/article/10.1186/s13012-017-0605-9. 10.1186/s13012-017-0605-9 PMC5480145 28637486

[23] Ethiopia moves to enhance AMR National Action Plan implementation through Public-Private Partnerships (PPPs). - WOAH - Africa [Internet]. [cited 2025 Aug 22]. Available from: https://rr-africa.woah.org/en/trainings/ethiopia-moves-to-enhance-amr-national-action-plan-implementation-through-public-private-partnerships-ppps/.

[24] Daniel Asfaw Erku, A. B. M. A. B. Inappropriate use of antibiotics among communities of Gondar town, Ethiopia: a threat to the development of antimicrobial resistance | Antimicrobial Resistance & Infection Control | Full Text [Internet]. 2017 [cited 2024 Dec 23]. Available from: https://aricjournal.biomedcentral.com/articles/10.1186/s13756-017-0272-2. 10.1186/s13756-017-0272-2 PMC5678763 29152233

[25] Full article. Risk Factors for Inappropriate Antimicrobial Therapy Among Patients with Hospital-Acquired Infection at Jimma Medical Center: A Prospective Observational Study [Internet]. [cited 2025 Jul 14]. Available from: https://www.tandfonline.com/doi/full/10.2147/IDR.S349358 10.2147/IDR.S349358 PMC8904264 35281573

[26] Pius, G. et al. Prevalence, determinants and knowledge of antibacterial self-medication: A cross sectional study in North-eastern Tanzania | PLOS One [Internet]. 2018 [cited 2025 Jul 1]. Available from: https://journals.plos.org/plosone/article?id=10.1371/journal.pone.0206623. 10.1371/journal.pone.0206623 PMC6209340 30379961

[27] Majekodunmi, A. O. et al. Through a gender lens: a scoping review of gendered experiences of AMR causes, burden and workforce in Nigeria. *Front. Glob Womens Health*. **6**, 1523901 (2025). 40475120 10.3389/fgwh.2025.1523901 PMC12137349

[28] Ramdas, N. et al. Knowledge, attitudes, motivations and expectations regarding antimicrobial use among community members seeking care at the primary healthcare level: a scoping review protocol. *BMJ Open.* **15** (1), e088769 (2025). 39894520 10.1136/bmjopen-2024-088769 PMC11792286

[29] Angela, M. et al. Psychological interventions to foster resilience in healthcare professionals - PMC [Internet]. 2020 [cited 2025 Jul 1]. Available from: https://pmc.ncbi.nlm.nih.gov/articles/PMC8121081/

[30] Kankanala, S. et al. Digital platform for prescription analytics and antibiotic surveillance in an outpatient department setup. *Indian J. Pharmacol.* **57** (1), 27–32 (2025). 40324828 10.4103/ijp.ijp_201_25 PMC12133053

[31] Almeman, A. The digital transformation in pharmacy: embracing online platforms and the cosmeceutical paradigm shift | Journal of Health, Population and Nutrition [Internet]. [cited 2025 Jul 2]. Available from: https://link.springer.com/article/10.1186/s41043-024-00550-2. 10.1186/s41043-024-00550-2 PMC11080122 38720390

[32] Burstein, V. R. et al. Communication interventions to promote the public’s awareness of antibiotics: a systematic review. *BMC Public. Health*. **19** (1), 899 (2019). 31286948 10.1186/s12889-019-7258-3 PMC6615171

[33] Mathew, P., Sivaraman, S. & Chandy, S. Communication strategies for improving public awareness on appropriate antibiotic use: bridging a vital gap for action on antibiotic resistance. *J. Fam Med. Prim. Care*. **8** (6), 1867–1871 (2019). 10.4103/jfmpc.jfmpc_263_19 PMC6618197 31334147

[34] Maduko, W. et al. Public-targeted interventions addressing antimicrobial resistance and antibiotic use in Sub-Saharan africa: a scoping review. *BMJ Glob Health*. **10** (3), e017455 (2025). 40118465 10.1136/bmjgh-2024-017455 PMC11931924

[35] Khan, M. S. et al. Caught in each other’s traps: factors perpetuating incentive-linked prescribing deals between physicians and the pharmaceutical industry. *Int. J. Health Policy Manag* . **13**, 8213 (2024). 10.34172/ijhpm.2024.8213 PMC11270611 38618843

[36] Jackson, T. H. Pharmacy value-based incentive programs: an evaluation of health plan strategies, pharmacist attitudes, and financial impact on retail stores (2019).

[37] Godman, B. et al. Strategies to improve antimicrobial utilization with a special focus on developing countries. *Life* **11** (6), 528 (2021). 34200116 10.3390/life11060528 PMC8229985

