# Supplementary material for: Exploring behavioral determinants of antimicrobial dispensing in drug retail outlets of Addis Ababa, Ethiopia: a mixed methods study
Source: Sci Rep. 2025 Oct 17;15:36423. doi: 10.1038/s41598-025-20558-w (PMC12534449; doi:10.1038/s41598-025-20558-w)
Supplement: Supplementary file 1 — Supplementary Material 1 [file 41598_2025_20558_MOESM1_ESM.docx]

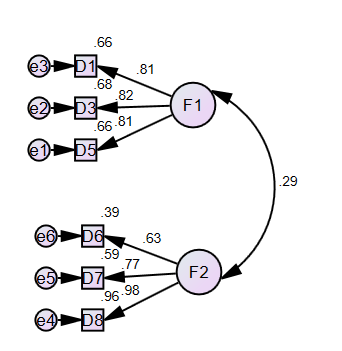


Fig. CFA output of dispensing behavior

**Normality assumption tests**


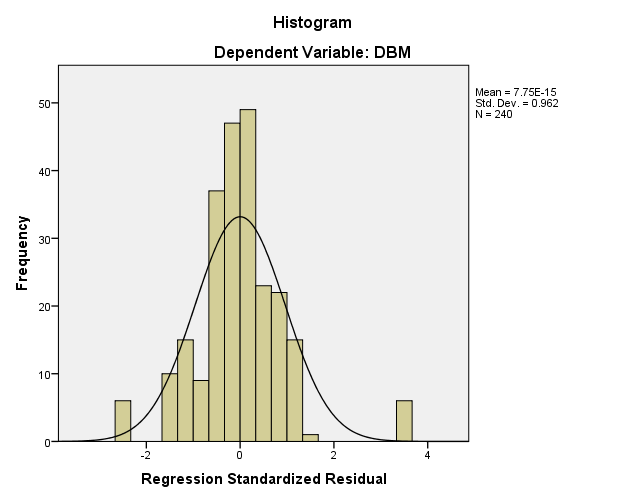


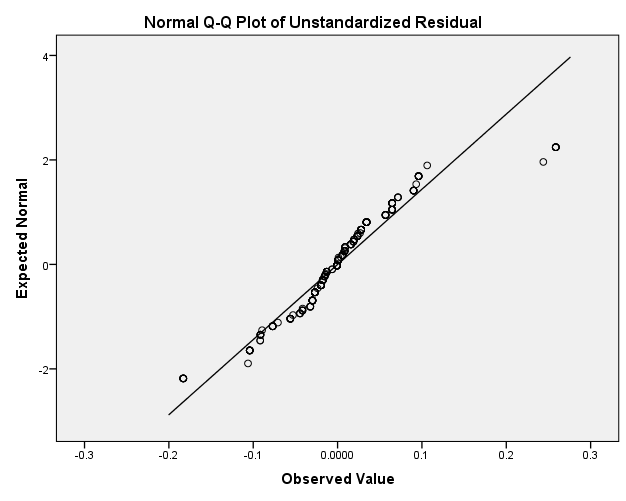


| **Model Summary^c^** | | | | | | | | | | |
| --- | --- | --- | --- | --- | --- | --- | --- | --- | --- | --- |
| Model | R | R Square | Adjusted R Square | Std. Error of the Estimate | Change Statistics | | | | | Durbin-Watson |
|  |  |  |  |  | R Square Change | F Change | df1 | df2 | Sig. F Change |  |
| 1 | .551^a^ | .304 | .289 | .22943 | .304 | 20.429 | 5 | 234 | .000 |  |
| 2 | .787^b^ | .620 | .607 | .17060 | .316 | 64.070 | 3 | 231 | .000 | 1.983 |
| a. Predictors: (Constant), Year of experience , Practice setting , Gend_Dum, Level of education , Age | | | | | | | | | | |
| b. Predictors: (Constant), Year of experience , Practice setting , Gend_Dum, Level of education , Age, REGR factor score 1 for analysis 2, REGR factor score 2 for analysis 2, REGR factor score 3 for analysis 2 | | | | | | | | | | |
| c. Dependent Variable: DBM | | | | | | | | | | |

| **ANOVA^a^** | | | | | | |
| --- | --- | --- | --- | --- | --- | --- |
| Model | | Sum of Squares | df | Mean Square | F | Sig. |
| 1 | Regression | 5.377 | 5 | 1.075 | 20.429 | .000^b^ |
|  | Residual | 12.317 | 234 | .053 |  |  |
|  | Total | 17.693 | 239 |  |  |  |
| 2 | Regression | 10.971 | 8 | 1.371 | 47.119 | .000^c^ |
|  | Residual | 6.723 | 231 | .029 |  |  |
|  | Total | 17.693 | 239 |  |  |  |
| a. Dependent Variable: DBM | | | | | | |
| b. Predictors: (Constant), Year of experience , Practice setting , Gend_Dum, Level of education , Age | | | | | | |
| c. Predictors: (Constant), Year of experience , Practice setting , Gend_Dum, Level of education , Age, REGR factor score 1 for analysis 2, REGR factor score 2 for analysis 2, REGR factor score 3 for analysis 2 | | | | | | |
